# Coupled Antigen and BLIMP1 Asymmetric Division With a Large Segregation Between Daughter Cells Recapitulates the Temporal Transition From Memory B Cells to Plasma Cells and a DZ-to-LZ Ratio in the Germinal Center

**DOI:** 10.3389/fimmu.2021.716240

**Published:** 2021-08-17

**Authors:** Elena Merino Tejero, Danial Lashgari, Rodrigo García-Valiente, Jiaojiao He, Philippe A. Robert, Michael Meyer-Hermann, Jeroen E. J. Guikema, Huub Hoefsloot, Antoine H. C. van Kampen

**Affiliations:** ^1^Bioinformatics Laboratory, Epidemiology and Data Science, Amsterdam Public Health Research Institute, Amsterdam Institute for Infection and Immunity, Amsterdam, Netherlands; ^2^Bioinformatics and Systems Biology, Vrije Universiteit Amsterdam, Amsterdam, Netherlands; ^3^Bioinformatics and Systems Biology, Institute for Life Sciences, University of Amsterdam, Amsterdam, Netherlands; ^4^Department for Systems Immunology and Braunschweig Integrated Centre of Systems Biology, Helmholtz Centre for Infection Research, Braunschweig, Germany; ^5^Institute for Biochemistry, Biotechnology and Bioinformatics, Technische Universität Braunschweig, Braunschweig, Germany; ^6^Department of Pathology, Lymphoma and Myeloma Center Amsterdam (LYMMCARE), Amsterdam University Medical Centers, Amsterdam, Netherlands; ^7^Biosystems Data Analysis, Swammerdam Institute for Life Sciences, University of Amsterdam, Amsterdam, Netherlands

**Keywords:** asymmetric division, germinal center, plasma cell differentiation, multiscale modeling, agent-based modeling

## Abstract

Memory B cells and antibody-secreting plasma cells are generated within germinal centers during affinity maturation in which B-cell proliferation, selection, differentiation, and self-renewal play important roles. The mechanisms behind memory B cell and plasma cell differentiation in germinal centers are not well understood. However, it has been suggested that cell fate is (partially) determined by asymmetric cell division, which involves the unequal distribution of cellular components to both daughter cells. To investigate what level and/or probability of asymmetric segregation of several fate determinant molecules, such as the antigen and transcription factors (BCL6, IRF4, and BLIMP1) recapitulates the temporal switch and DZ-to-LZ ratio in the germinal center, we implemented a multiscale model that combines a core gene regulatory network for plasma cell differentiation with a model describing the cellular interactions and dynamics in the germinal center. Our simulations show that BLIMP1 driven plasma cell differentiation together with coupled asymmetric division of antigen and BLIMP1 with a large segregation between the daughter cells results in a germinal center DZ-to-LZ ratio and a temporal switch from memory B cells to plasma cells that have been observed in experiments.

## Introduction

Memory B cells (MBCs) and antibody-secreting plasma cells (PCs) are generated within germinal centers (GCs) during affinity maturation in which B-cell proliferation, selection, differentiation, and self-renewal play important roles in the GC reaction (1). Positive selection of B cells is facilitated by collecting antigen (Ag) presented by follicular dendritic cells (FDCs) and subsequent engagement in T follicular helper (Tfh) cells contacts. B cells with higher-affinity receptors (BcRs) are thought to receive more help from Tfh cells due to increased presentation of pMHCII on their surface. Selected B cells recycle to the dark zone (DZ) to further divide and differentiate as output cells (OCs) or to enter a next cycle of selection (recycling).

The mechanisms behind MBC and PC differentiation into OCs from GCs are not well understood. However, in other systems, such as *Drosophila*, it has been suggested that cell fate is (partially) determined by asymmetric cell division, which involves the unequal distribution of cellular components to both daughter cells (2). Another study exclusively analyzed the distribution of Ag in *in vivo* and *in vitro* mouse B cells showing that accumulated Ag is maintained in a polarized distribution prior to the division in approximately 72% of the B cells and that this polarization is maintained during cell division resulting in an asymmetric division of Ag over both daughter cells (3). The daughter cell that receives more Ag as a result of asymmetric division was postulated to be more efficient in receiving T cell help, both at the B–T cell border and in the GC, which may affect cell fate (3). In the same issue, it was argued and shown by computational modeling that asymmetric division may largely affect the production of PCs (4). Later, a more comprehensive computational model of the GC reaction predicted that asymmetric division of Ag might codetermine B-cell fate, since inclusion of this mechanism resulted in GC transzone migration rates and DZ-to-LZ ratio in agreement with experimental data (5, 6). In addition to asymmetric Ag division, *in vitro* studies have shown that other B-cell fate-altering molecules, such as transcriptional regulator B-cell lymphoma 6 (BCL6) and the receptor for interleukin-21 (IL-21R), segregate asymmetrically in approximately 44% of mitotic GC B cells (7). In contrast, IRF4 was mostly symmetrically distributed (11% asymmetry comparable to tubulin). The same study suggested that CD40 signaling facilitates TF asymmetry by providing polarity cues to B cells. However, other polarity cues [e.g., cell–cell contacts (8)], TFs [e.g., BLIMP1 transcription (9)], and signaling pathways [e.g., nuclear factor kappa B (Nf-kB)] may drive asymmetric division and/or B-cell fate.

Regardless of the mechanism, asymmetric division has been shown to result in daughter cells with unequal amounts of Ag and/or TF. The amount of segregation seems to vary for different TFs, and this might be dependent on polarity cues, signaling pathways and strength, and/or stochastic events. We hypothesized that (the level of) Ag and TF (BCL6, IRF4, BLIMP1) segregation affects GC dynamics and B-cell fate in different ways or to different extents. To test this hypothesis, we implemented a multiscale model (MSM) that combines a core gene regulatory network for B cell of PC differentiation with a model describing the cellular interactions and dynamics in the GC.

Our simulations show that BLIMP1-driven PC differentiation coupled to asymmetric division of Ag and BLIMP1 with a large segregation between the daughter cells results in a GC transzone migration and a temporal switch from MBCs to PCs that are both observed in experiments (6, 10). Consequently, these computational results prompt for more direct experiments aimed to verify or falsify this mechanism for PC differentiation.

## Methods

### Multiscale Model

To enable the investigation of cellular and molecular mechanisms involved in PC differentiation, we recently developed a multiscale model (MSM) (11) that integrates an agent-based model (ABM) of the GC reaction (5) with a gene regulatory network (GRN) involved in PC differentiation (12). We slightly modified this model to investigate the effect of asymmetric Ag and TF division. In brief, the ABM contains the main processes that take place in the GC reaction, which lasts for 21 days (504 h). B cells at the centroblast (CB) state divide in the DZ while accumulating SHMs in their BcR. They then differentiate to CCs and migrate to the LZ where they may encounter FDCs and Tfh cells. FDCs carry Ag in their membrane, which is internalized by CCs when in contact with an affinity-dependent rate. This provides CCs with survival signals that temporarily rescue them from apoptosis and allow them to undergo further encounter(s) with Tfh cells. CCs with higher internalized Ag, thus higher affinity for the Ag, will outcompete other CCs with less internalized Ag. CCs are then fully rescued from apoptosis and recycle back to the DZ as CBs. Recycled CBs further divide asymmetrically in 72% of the cases where all of the internalized Ag goes to one of the daughter cells. The GRN of PC differentiation comprises three TFs (BLIMP1, BCL6, and IRF4) that regulate each other and are affected by upstream BcR and CD40 signals. BCL6 is involved in maintaining GC B-cell phenotype, while IRF4 and BLIMP1 promote PC differentiation and exit from the GC. Initial TF concentration in founder CBs were based on microarray data (12) and defined as follows (BLIMP1 = 0, BCL6 = 5, and IRF4 = 0) to achieve the high BCL6 and low BLIMP1 and IRF4 steady state. CCs receive signals through BcR and CD40 respectively when in contact with FDCs or Tfh cells. In the model, BcR signal strength is assumed to be constant, while CD40 signal strength depends on affinity, which can range between 0 and 1, and determines the B-cell fate. The GRN is a bistable system with one state (BCL6 high, BLIMP1/IRF4 low) being the intracellular state of CBs, CCs, and MBCs and a second state (BLIMP1/IRF4 high, BCL6 low) representing the intracellular state of PCs. After dividing, recycled CBs that inherited all of the internalized Ag, and/or are in BLIMP1 high state, differentiate to OCs, either MBCs or PCs, while the remaining CBs differentiate to CCs and stay in the GC. Ag in the CCs is removed, giving no advantage in further rounds of selection.

### Definition of Output Cells and Memory *Versus* PC Differentiation Fate

**Figure 1:**
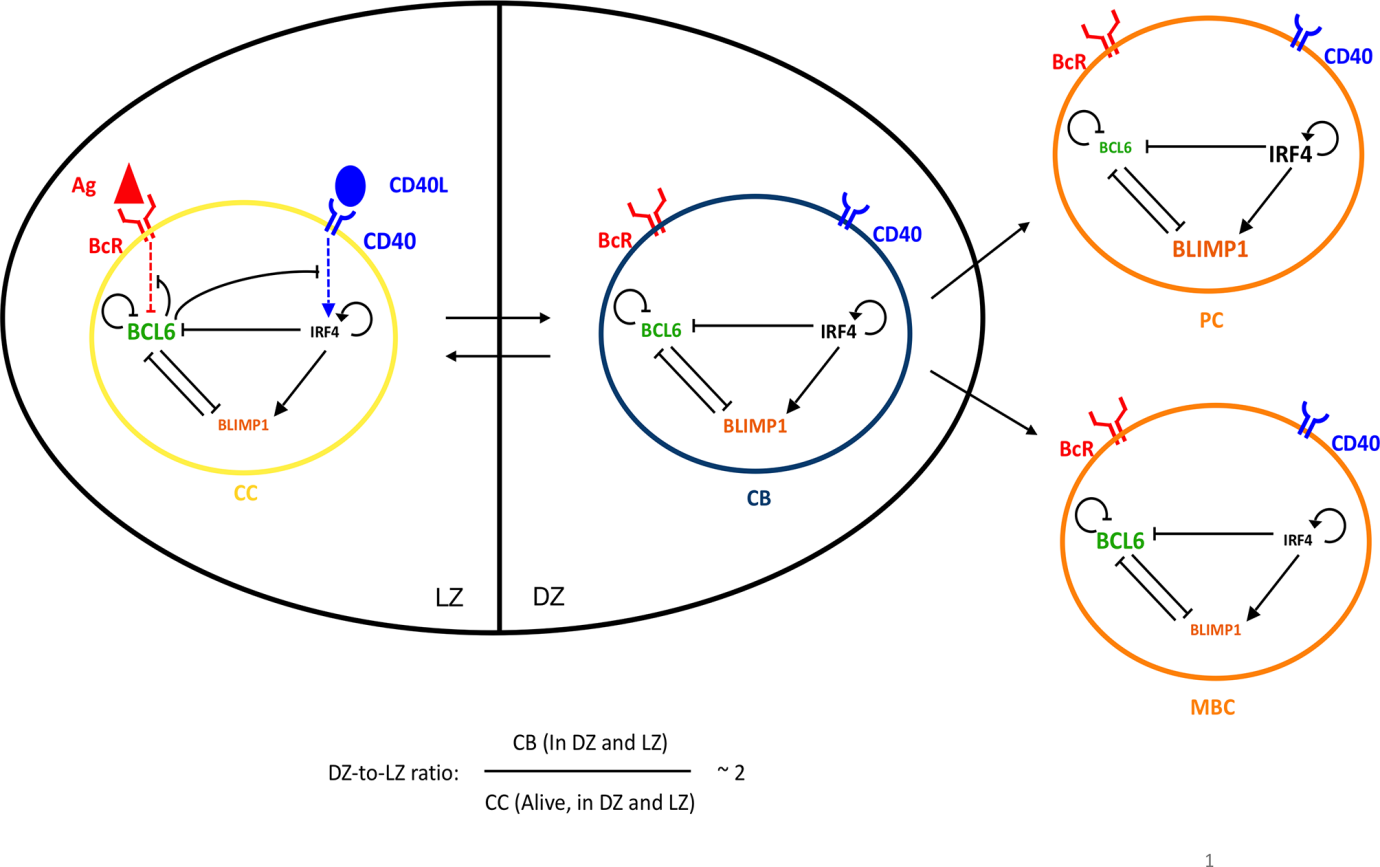
Four GC B cells representing the PC differentiation process: CC (yellow), CB (blue), PC (orange), and MBC (orange). CBs are mainly present in the DZ and CCs in the LZ, while PCs and MBCs are mainly generated in the DZ and then exit the GC. Transition between CBs and CCs is reversible, while the transition between CBs and PCs or MBCs is irreversible. The DZ-to-LZ ratio is the ratio of CBs to non-apoptotic CCs present in both zones and fluctuates around 2. An intracellular GRN comprising three TFs is embedded in each B cell: BCL6 (green), IRF4 (black), and BLIMP1 (orange). The size of each TF represents the expression levels in the cell state. The CC BcR may bind to Ag (red) or the CD40L (blue) when receiving T-cell help, resulting in BcR and CD40 signaling, respectively, which changes the state of the network. Arrows between cells represent transition. Arrows between TFs, BcR, and/or CD40 indicate activation. Bar-headed lines denote inhibition.

**Table 1** shows the cell type definition based on Ag status and BLIMP1 level. Recycled CBs that finish dividing may differentiate to PCs at any time of the GC reaction (**Figure 1**) when BLIMP1 reaches the differentiation threshold (≥8.10^−8^M) and become BLIMP1+ irrespective of its Ag status, and, consequently, PCs may either be Ag+ or Ag−. BLIMP1+ cells that are not (yet) OCs are annotated as PB (Ag+ or Ag−). Ag+/BLIMP1− OCs are considered to be MBCs. This definition correctly recapitulates the MBC dynamics as described in Weisel and coworkers (10). Finally, Ag-/BLIMP1− CBs stay in the GC and recycle back to the LZ as CCs.

**Table 1:** Definition of OCs (PCs and MBCs) in terms of Ag status and BLIMP1 level.

PC	Ag+/BLIMP1+	Ag−/BLIMP1+
MBC	Ag+/BLIMP1−	

### Modeling of Asymmetric Division

In the current model, we do not distinguish between different mechanisms that lead to asymmetry but only assume that Ag and TFs (BCL6, IRF4, BLIMP1) can be unequally distributed between the two daughter cells. Asymmetric division is parameterized by a probability (P) of asymmetric division and a polarity level (L) representing the extent of asymmetry. Following experimental observations from Thaunat and coworkers, we set the probability for asymmetric division of Ag to either P_Ag_ = 0.0 or P_Ag_ = 0.72 (3). The same study showed that Ag division can happened both symmetrically and asymmetrically, which is why we did not further investigate asymmetric Ag probabilities of 100%. Consequently, in 0% or 72% of the cell divisions, the Ag is distributed asymmetrically over the daughter cells. The probability of asymmetric division for TFs is unknown, and, therefore, we used three different probabilities: P_TF_ = 0.0, P_TF_ = 0.72, or P_TF_ = 1.0. Consequently, in 0%, 72%, or 100% of the cell divisions, the TFs are distributed asymmetrically over the daughter cells. In the current model, when the Ag and TFs are asymmetrically distributed in the same division, high Ag and TF polarity levels are directed towards the same daughter cell. Nevertheless, in this study, we are interested in simulating the effect of simultaneous asymmetric division of Ag and TFs.

The polarity level (L_Ag_ and L_TF_) of asymmetry represents the concentration of Ag and TFs in one daughter cell expressed as the fraction of Ag and TFs in the parent cell; the second daughter cell, by definition, assumes a concentration of 1-polarity. Consequently, a polarity level of L = 0.5 represents symmetric division (the concentration of Ag and TFs in each daughter cell is 50% of the parent cell). An asymmetric division probability P = 0.0, by definition, corresponds to a polarity level (L = 0.5). A polarity level of L = 1.0 results in one daughter cell that has taken all Ag and/or TFs from the parent cell, while the other daughter cell will receive none. In the simulations, the TFs may segregate with a different polarity levels (L_BLIMP1_, L_BCL6_, L_IRF4_).

### Simulations

We performed two sets of GC simulations. In the first set of nine simulations (**Table 2**), the TFs cosegregate with equal polarity levels, while in the second set of 27 simulations (**Table 3**), the TFs may cosegregate with different polarity levels. Simulation 3 from the first set (**Table 2**) is considered the reference simulation in which there is asymmetric division of Ag (P_Ag_) but always symmetric division of TFs. We consider this simulation as the reference since in the original LEDA model, no TFs were modeled, while asymmetric Ag division showed to result in a correct DZ-to-LZ ratio. The DZ-to-LZ ratio was calculated as the ratio of CBs to non-apoptotic CCs present in both zones (**Figure 1**). Since Simulations 1–3 from the second set of 27 simulations (**Table 3**) were the only cases to show differences in the MBC and PC dynamics, we repeated these simulations 15 times with different random seeds. **Supplementary Figures 1–3** show the results from these repetitions and demonstrate that there is a limited variability in the temporal dynamics. Therefore, we did not repeat the other simulations, since these are expected to give a similar amount of variation.

**Table 2:** Simulated asymmetry of TF concentrations (polarity level L_TF_) in daughter cells after division.

Simulation	Description	Mode Ag division	
		Asymmetric	Symmetric
		TF polarity level (L_TF_)	
1	(i) Symmetric Ag and TF division (P_Ag_ = P_TF_ = 0)	N.A.	0.5
2	(ii) Symmetric Ag division and asymmetric TF division (P_Ag_ = 0, P_TF_ = 0.72)	N.A.	1.0
3	(iii) Symmetric TF division and asymmetric Ag division (reference; P_Ag_ = 0.72, P_TF_ = 0)	0.5	0.5
4	(iv) Asymmetric TF division only if mode of Ag division is asymmetric (coupled asymmetric division; P_Ag_ = P_TF_ = 0.72)	1.0	0.5
5		0.9	0.5
6		0.75	0.5
7	(v) Always asymmetric TF division regardless of mode of Ag division (uncoupled asymmetric division; P_Ag_ = 0.72, P_TF_ = 1.0)	1.0	1.0
8		0.9	0.9
9		0.75	0.75

When mode of Ag division is asymmetric the probability and polarity level are P_Ag_ = 0.72; L_Ag_ = 1.0; otherwise, these are set to (P_Ag_ = 0.0; L_Ag_ = 0.5) for symmetric Ag division. In these nine simulations, BCL6, IRF4, and BLIMP1 are cosegregated.

**Table 3:** Simulated asymmetry of TFs concentrations (P_TF_ = 0.72; polarity levels L_BLIMP1_, L_IRF4_, and L_BCL6_) in daughter cells after asymmetric division.

	Mode Ag division			
	Asymmetric			Symmetric
	Polarity level (L_TF_)			
Simulations	BLIMP1 (L_BLIMP1_)	IRF4(L_IRF4_)	BCL6 (L_BCL6_)	BLIMP1, IRF4, BCL6(L_BLIMP1_ = L_IRF4_ − L_BCL6_)
1–3		1.0	1.0	0.5
4–6		0.9	1.0	0.5
7–9		0.75	1.0	0.5
10–12	1.0 0.75 0.9	1.0	0.9	0.5
13–15		0.9	0.9	0.5
16–18		0.75	0.9	0.5
19–21		1.0	0.75	0.5
22–24		0.9	0.75	0.5
25–27		0.75	0.75	0.5

TFs divide asymmetrically if Ag divides asymmetrically (P_Ag_ = P_TF_ = 0.72; L_Ag_ = 1.0). In these 27 simulations, BCL6, IRF4, and BLIMP1 do not always cosegregate with same polarity levels.

**Figure 2:**
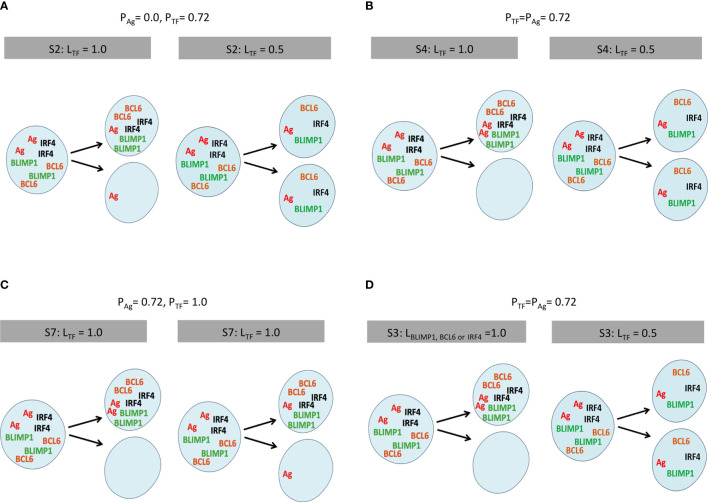
Scheme of internalized Ag and TF division patterns modeled in a selection of simulations (**Tables 2**, **3**). **(A)** Simulation 2, symmetric Ag and asymmetric TF distribution (L_TF_ = 1.0). **(B)** Simulation 4, coupled asymmetric division (L_TF_ = 1.0). **(C)** Simulation 7, uncoupled asymmetric division (L_TF_ = 1.0), and **(D)** simulation 3, partial asymmetric co-segregation of TFs and Ag (P_TF_ = P_Ag_ = 0.72; L_Ag_ = 1.0) while varying the level of BLIMP1 (L_BLIMP1_ = 1.0). Internalized Ag (red) and TF (orange, green, black) are shown in the parent and two daughter cells. The probability and polarity levels are shown in the gray box.

In the first set of simulations, we studied different combinations of Ag and TF (a)symmetric division (**Table 2**). In these simulations, the TFs are cosegregated over the daughter cells according to the polarity levels (L_TF_) shown in **Table 2**. The polarity level for the asymmetric Ag division is always L_Ag_ = 1.0. These nine simulations represent five scenarios: (i) TFs and Ag divide symmetrically (P_TF_ = P_Ag_ = 0.0); (ii) TFs divide asymmetrically with probability P_TF_ = 0.72, while Ag always divides symmetrically (P_Ag_ = 0.0; **Figure 2A**); (iii) TFs divide symmetrically (P_TF_ = 0.0), while Ag can divide asymmetrically (P_Ag_ = 0.72; reference); (iv) TFs divide asymmetrically (P_TF_ = 0.72) only when Ag divides asymmetric (P_Ag_ = 1.0; **Figure 2B**); and (5) TFs always divide asymmetrically (P_TF_ = 1.0), while Ag divides asymmetrically with probability P_Ag_ = 0.72 (**Figure 2C**).

In the second set of 27 simulations, the Ag is distributed asymmetrically in 72% of the recycled B-cell divisions (P_Ag_ = 0.72, L_Ag_ = 1.0; **Table 3**), since it was previously shown that this results in transzone migration rates in better agreement with experimental data (5). In these simulations, the TFs cosegregate with the Ag, since they only divide asymmetrically when the Ag divides asymmetrically (P_Ag_ = P_TF_ = 0.72). Moreover, TFs segregate with different polarity levels (L_BLIMP1_, L_BCL6_, L_IRF4_) as shown in **Figure 2D**.

### Simulation of Gene Regulatory Network

To facilitate the interpretation of the MSM, we additionally performed a set of GRN simulations to model TF dynamics. For these simulations, initial TF concentration of the mother cell was conceptually chosen to simulate an extreme condition of our MSM in which a mother PB, at the low BCL6 and high BLIMP1 and IRF4 steady state, underwent the last division before becoming a PC and exiting the GC. Subsequently, asymmetric division of the parent PB was simulated with the different combinations of L_TFs_ for the first set of simulations (**Table 2**). For the second set of simulations, we investigated representative L_BLIMP1_, L_BCL6_, and L_IRF4_ combinations (i.e., simulations 1–4, 7, 10, 19; **Table 3**). At the start of the simulation, we defined the concentrations of BLIMP1, BCL6, and IRF4 according to the polarity levels and, subsequently, simulate until a steady state was reached. This allowed us to determine if despite the concentration reduction, BLIMP1 concentration returned to its high level steady state (PC phenotype). Since we were simulating TF dynamics of CBs that do not interact with Ag presented by FDCs nor with Tfh cells, we set the CD40 and BcR signals to 0.

## Results

### Symmetric TF and Ag Division

We first aimed to gain insight in the contribution of asymmetric division on GC dynamics and OCs. Therefore, we simulated the GC reaction without asymmetric Ag and TFs division (P_TF_ = P_Ag_ = 0.0, L_TF_ = L_Ag_ = 0.5; simulation 1, **Table 2**).

**Figure 3:**
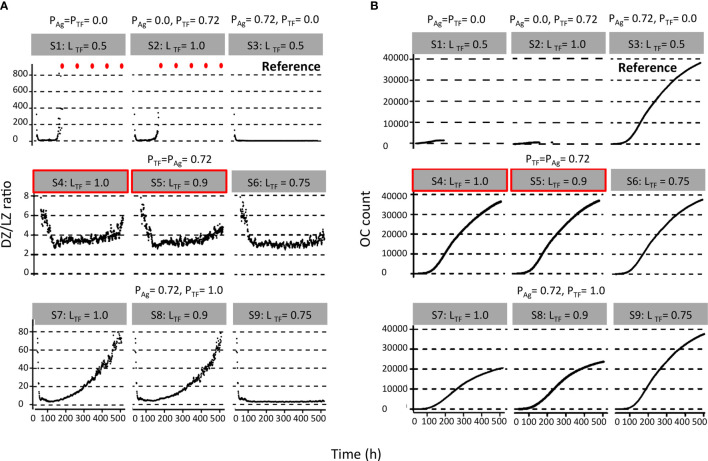
Results from first set of simulations (**Table 2**). **(A)** DZ-to-LZ ratio and **(B)** accumulated OCs during the GC reaction. The probability of asymmetric division (P) is indicated above the gray box, and simulation number and polarity levels (L) are shown in the gray box. Red dots indicate DZ-to-LZ ratio values of infinity. First row of plots corresponds to (left column) symmetric division of Ag and TFs, (middle column) symmetric division of Ag and asymmetric division of TFs, and (right column) symmetric division of TFs and asymmetric division of Ag. Second row of plots corresponds to asymmetric TF division only if mode of Ag division is asymmetric. Red boxes indicate parameters that are closer to biological results. Third row of plots corresponds to always asymmetric TF division regardless of mode of Ag division.

**Figure 4:**
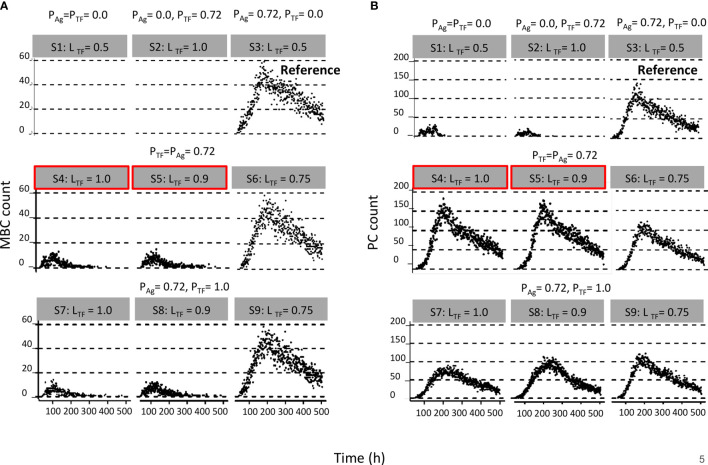
Results from first set of simulations (**Table 2**). **(A)** Relative MBC and **(B)** PC count during the GC. The probability of asymmetric division (P) is indicated above the gray box and simulation number and polarity levels (L) are shown in the gray box. First row of plots corresponds to (left column) symmetric division of Ag and TFs, (middle column) symmetric division of Ag and asymmetric division of TFs, and (right column) symmetric division of TFs and asymmetric division of Ag. Second row of plots corresponds to asymmetric TF division only if mode of Ag division is asymmetric. Red boxes indicate parameters that are closer to biological results. Third row of plots corresponds to always asymmetric TF division regardless of mode of Ag division.

We found a DZ-to-LZ ratio that initially fluctuated between 5 and 15 and then increased to values up to 800 or the ratio became infinite due to low or zero CC counts, respectively (**Figure 3A**), strongly contradicting experimentally observed DZ-to-LZ ratio of 2. This is explained by a lack of recycled CBs without retained Ag, which led to no differentiation to CC state and a premature termination of the GC reaction. Thus, the number of accumulated OCs reached 1,417 cells at the end of the GC reaction (**Table 4** and **Figure 3B**). No MBCs were produced (**Figure 4A**), and all OCs were PCs (**Figure 4B**) due to the lack of Ag+ cells. Furthermore, 87% of PCs were generated within the first 6 days of the GC reaction, which contradicts a temporal switch from MBCs to PCs (**Supplementary Figure 3**).

**Table 4:** Number of OCs at day 21 originating from the first set of simulations (**Table 2**).

	**P_Ag_**	0.0		0.72						
	**P_TF_**	0.0	0.72	0.0	0.72			1.0		
	**L_TF_**	0.5	1.0	0.5	1.0	0.9	0.75	1.0	0.9	0.75
	**Simulation**	**1**	**2**	**3 (ref)**	**4**	**5**	**6**	**7**	**8**	**9**
**OCs**	**PCs**	1,417	759	25,246	35,359	35,791	25,792	19,784	22,755	24,821
	**MBCs**	0	0	12,886	1,094	1106	12,704	824	948	12,787
	**Total**	1,417	759	38,132	36,453	36,898	38,496	20,608	23,703	37,608

### Asymmetric TF Division and Symmetric Ag Division

**Figure 5:**
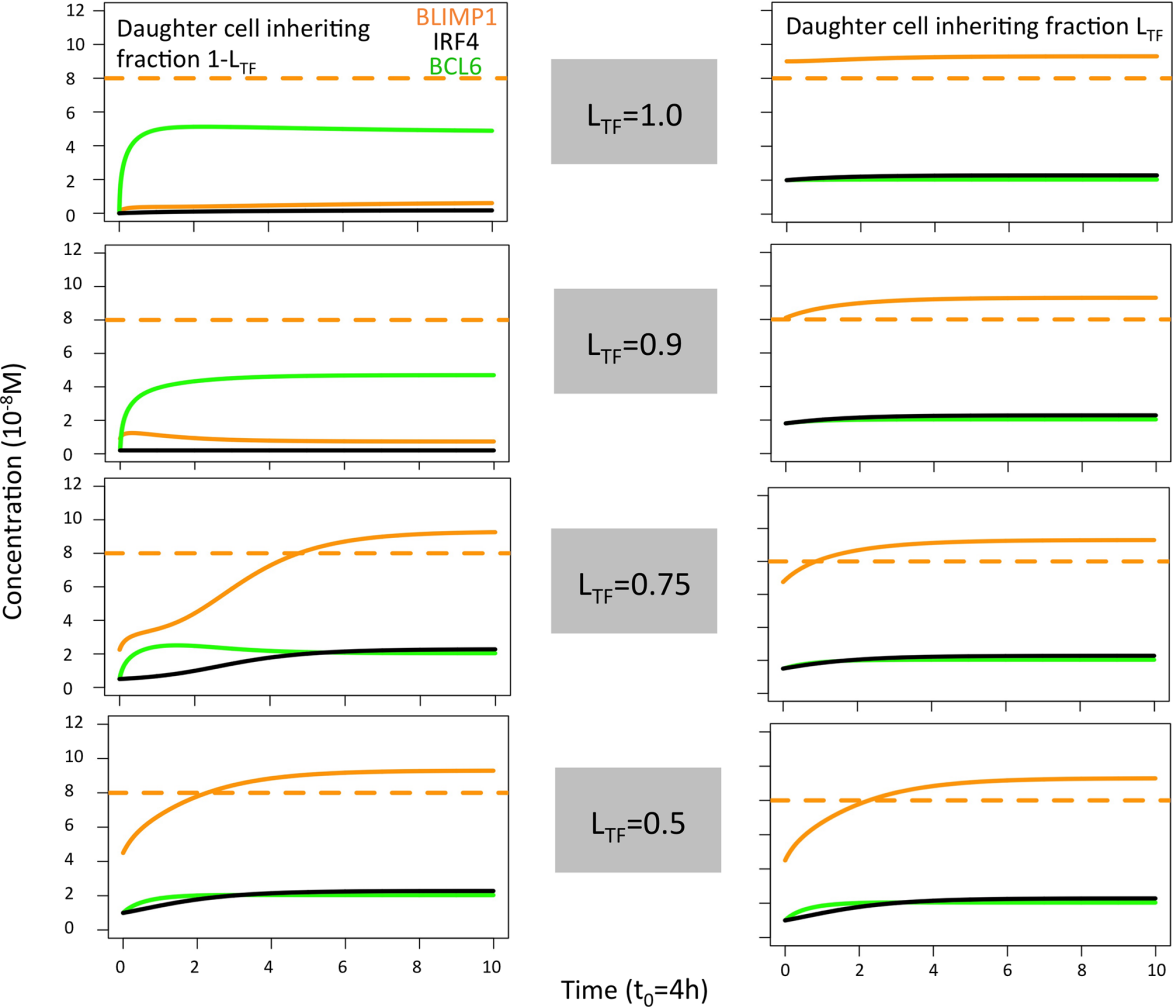
Solution curves based on the GRN (ODE model) for BLIMP1 (orange), BCL6 (green), and IRF4 (black) in two daughter cells. The initial TF concentrations were based on the concentration of the parent cell (BLIMP1 = 8, BCL6 = 2, IRF4 = 2) and the different polarity levels (L_TF_ = 1.0, L_TF_ = 0.9, L_TF_ = 0.75, and L_TF_ = 0.5; **Table 2**).

Next, we aimed to establish the effect of asymmetric TF division while keeping symmetric Ag division (simulation 2, **Table 2**; P_Ag_ = 0.0, P_TF_ = 0.72, L_Ag_ = 0.5, L_TF_ = 1.0). Again, we find that the DZ-to-LZ ratio initially fluctuated between 5 and 15 and then increased until 400 or was infinite since no CCs were produced (**Figure 3A**) strongly contradicting experimentally observed DZ-to-LZ ratio of 2. In addition, the number of accumulated OCs reached 759 cells at the end of the GC reaction, none of them being MBCs (**Table 4**; **Figures 3B**, **4**) Furthermore, 92% of PCs were generated within the first 6 days of the GC reaction (**Supplementary Figure 3**) again contradicting a temporal switch. Finally, asymmetric TF division led to approximately a twofold decrease in PC production compared to symmetric TF division (simulation 1) as shown in **Table 4**. This could be explained by analyzing the TF dynamics in isolation (**Figure 5**). Extreme TF polarity levels promoted the production of a daughter B cell in the low BLIMP1 state and another one in the high BLIMP1 state, yet symmetric TF polarities promoted the production of both daughter B cells in the high BLIMP1 state. We conclude that asymmetric division of TF only does not result in expected GC dynamics while also the number of OCs remains 50-fold lower than in the reference simulation.

### Symmetric TFs Division and Asymmetric Ag Division (Reference)

We questioned whether or not symmetric cosegregation of TFs with asymmetric Ag division had an effect on GC B-cell dynamics (P_TF_ = 0.0, L_TF_ = 0.5, P_Ag_ = 0.72, L_Ag_ = 1.0; simulation 3, **Table 2**). We found the DZ-to-LZ ratio fluctuating between 2 and 4 (**Figure 3A**). This was a maximum of 2-fold increase in DZ-to-LZ ratio compared to previous observations of 2 (6) and similar to the affinity-based CD40 signaling simulation (Scenario 2) discussed in (11). The number of accumulated OCs reached 38,132 cells at the end of the GC reaction (**Table 4** and **Figure 3B**) of which 12,886 were MBCs (**Figure 4A**) and 25,246 were PCs (**Figure 4B**). Furthermore, MBCs were generated throughout the GC reaction, and 90% of PCs were generated after the peak (day 6) of the GC reaction (**Supplementary Figure 3**). We conclude that asymmetric Ag division is largely responsible for obtaining a DZ-to-LZ ratio close to experimental observations. Asymmetric TF division is not required. Asymmetric Ag division also re-establishes a larger number of OCs, but no temporal switch is observed.

### Asymmetric TF Division Only if Mode of Ag Division Is Asymmetric (Coupled Asymmetric Division)

Next, we investigated a scenario (simulations 4–6, **Table 2**; P_Ag_ = P_TF_ = 0.72) that assumes that asymmetric TF and Ag division always happen simultaneously. Since we are mostly interested in the effect of the TFs, we assumed that in the case of asymmetric division, all Ag goes to a single daughter cell (L_Ag_ = 1.0) while we used different polarization levels for the TF (L_TF_ = 1.0, 0.9, and 0.75). All three simulations had similar DZ-to-LZ ratios and total number of OCs, which were also similar to the reference simulation (**Table 4** and **Figures 3A, B**). Nevertheless, low TF polarity levels showed approximately a 12-fold increase in MBCs, at the expense of PC output, compared to extreme TF polarity levels. Furthermore, low TF polarity levels showed similar MBC counts compared to the reference simulation (**Figure 4**). Interestingly, extreme TF polarity levels (L_TF_ = 1.0, 0.9) resulted in a temporal switch from MBCs to PCs, which was not the case for simulations with low TF polarity levels (L_TF_ = 0.75 nor L_TF_ = 0.5 in the reference simulation).

When analyzing the TF dynamics in the GRN, we found, as expected, that extreme TF polarity levels generated a high BLIMP1 state in one of the TF inheriting daughters (0 h, **Figure 5**) while leaving the other daughter B-cell in a low BLIMP1 state. Contrarily, low TF polarity levels promoted a slower progression to the high BLIMP1 state (4–8 h), which explains the increased number of MBCs (Ag+/BLIMP1−) in simulations 3 and 6. We conclude that simultaneous asymmetric division of Ag and TF results in DZ-to-LZ ratios similar to the reference simulation, but only extreme TF polarity levels resulted in a temporal switch.

### Always Asymmetric TF Division Regardless of Mode of Ag Division (Uncoupled Asymmetric Division)

Since there is no *a priori* reason to suggest that asymmetric Ag and TF division are coupled (simulations 4–6), we performed three additional simulations in which TF always divide asymmetrically (P_TF_ = 1.0, L_TF_ = 1.0, L_TF_ = 0.9, and L_TF_ = 0.75) regardless of the model of Ag division (P_Ag_ = 0.72, L_Ag_ = 1.0; simulations 7–9, **Table 2**). We found that for extreme TF polarity levels (L_TF_ = 1.0, 0.9), the DZ-to-LZ ratio progressively increased up to a value of 80, which meant a 40-fold increase compared to the reference simulation (**Figure 3A**). Contrarily, low TF polarity levels (L_TF_ = 0.75) showed a DZ-to-LZ ratio that fluctuated between 2 and 4 similarly to the reference simulation (**Figure 3A**). Extreme TF polarity levels showed a 2-fold decrease in OC counts and a 12-fold increase in MBC counts compared to low TF polarity levels and the reference simulation (**Table 4** and **Figure 3B**). In extreme TF polarity levels, there was a 1.2-fold decrease in PC counts compared to low TF polarity levels and a 1.7-fold decrease in PC counts compared to simulations with coupled asymmetric Ag and TFs division (**Table 4** and **Figure 4**). While approximately 90% of PCs were generated after the peak (day 6) of the GC reaction for all TF polarity levels, low TF polarity levels produced MBCs during the entire GC reaction (**Supplementary Figure 4**). Thus, while low polarity levels resulted in similar DZ-to-LZ ratio and OC production as the reference simulation, it did not result in a temporal switch from MBCs to PCs.

The TF dynamics in the GRN, as described in the previous section (see above, **Figure 5**), explained the decreased OC count observed in simulations 7 and 8 compared to simulations 4–6 and 9. In addition, it could explain the similarity in OC count observed when comparing simulations 6 and 9.

We concluded that uncoupled Ag and TFs asymmetric division lead to a 40-fold increase in DZ-to-LZ ratios and a reduction in OC production for the extreme TF polarity levels. However, for these extreme polarities, a temporal switch is observed.

Collectively, the first set of simulations show that assuming that the decision for PC differentiation is fully based on BLIMP1 levels and that all TFs cosegregate during asymmetric division, then the simulated DZ-to-LZ ratio is close to those observed experimentally. Furthermore, a temporal switch from MBCs to PCs was only present in simulations with coupled Ag and TFs asymmetric division and extreme TF polarities L_TF._

### Coupled Ag and TFs Asymmetric Division With Different Polarity Levels for Individual TFs

From the first set of simulations (simulations 1–9, **Table 2**), we showed that coupled Ag and TFs asymmetric division with extreme TF polarity levels resulted in a DZ-to-LZ ratio that was similar to the reference simulation and a temporal switch. However, in these simulations, we assumed that BCL6, IRF4, and BLIMP1 always distributed in equal amounts (L_TF_) over the daughter cells. Based on previous research, this is unlikely (7, 8). Therefore, we performed 27 additional simulations (**Table 3**; P_TF_ = P_Ag_ = 0.72 and L_Ag_ = 1.0) in which TFs can be distributed in different amounts (L_BLIMP1,_ L_IRF4_, and L_BCL6_) to the daughter cells. In these simulations TFs are only asymmetrically distributed in case of asymmetric Ag division. For each simulation, we investigated the GC dynamics and OC production.

**Figure 6:**
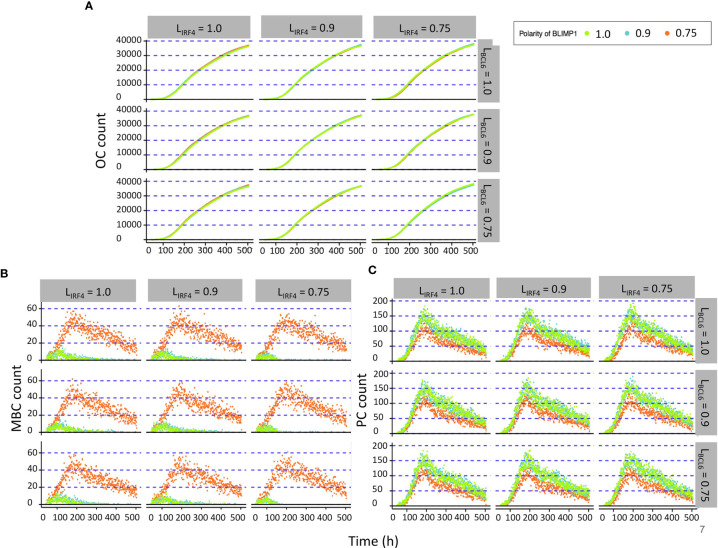
Results from the second set of simulations (**Table 3**). **(A)** Accumulated OCs, **(B)** relative MBC, and **(C)** PC count during the GC reaction. At the top of each panel column, the IFR4 polarity level is indicated. To the right of each panel row, the BCL6 polarity level is indicated. The colors indicate the different BLIMP1 polarity levels.

All simulations showed a DZ-to-LZ ratio that was similar to the reference simulation (data not shown). Furthermore, the number of OCs at the end of the GC reaction is similar for all 27 simulations (**Figure 6A**). **Figures 6B, C** show the number of MBCs and PCs produced for the 27 combinations of TF polarity levels. We observed that neither the polarity level of IRF4 nor BCL6 have a big influence on the number of OCs, MBCs, or PCs. However, there is a clear difference when comparing the extreme (L_BLIMP1_ = 1.0 and L_BLIMP1_ = 0.9; L_IRF4_ = L_BCL6_ = 1.0) and low (L_BLIMP1_ = 0.75; L_IRF4_ = L_BCL6_ = 1.0) BLIMP1 polarity levels. Low polarity levels resulted in a 12-fold increase in MBC counts and a 1.2-fold decrease in PC counts (**Supplementary Figures 5–7**).

**Figure 7:**
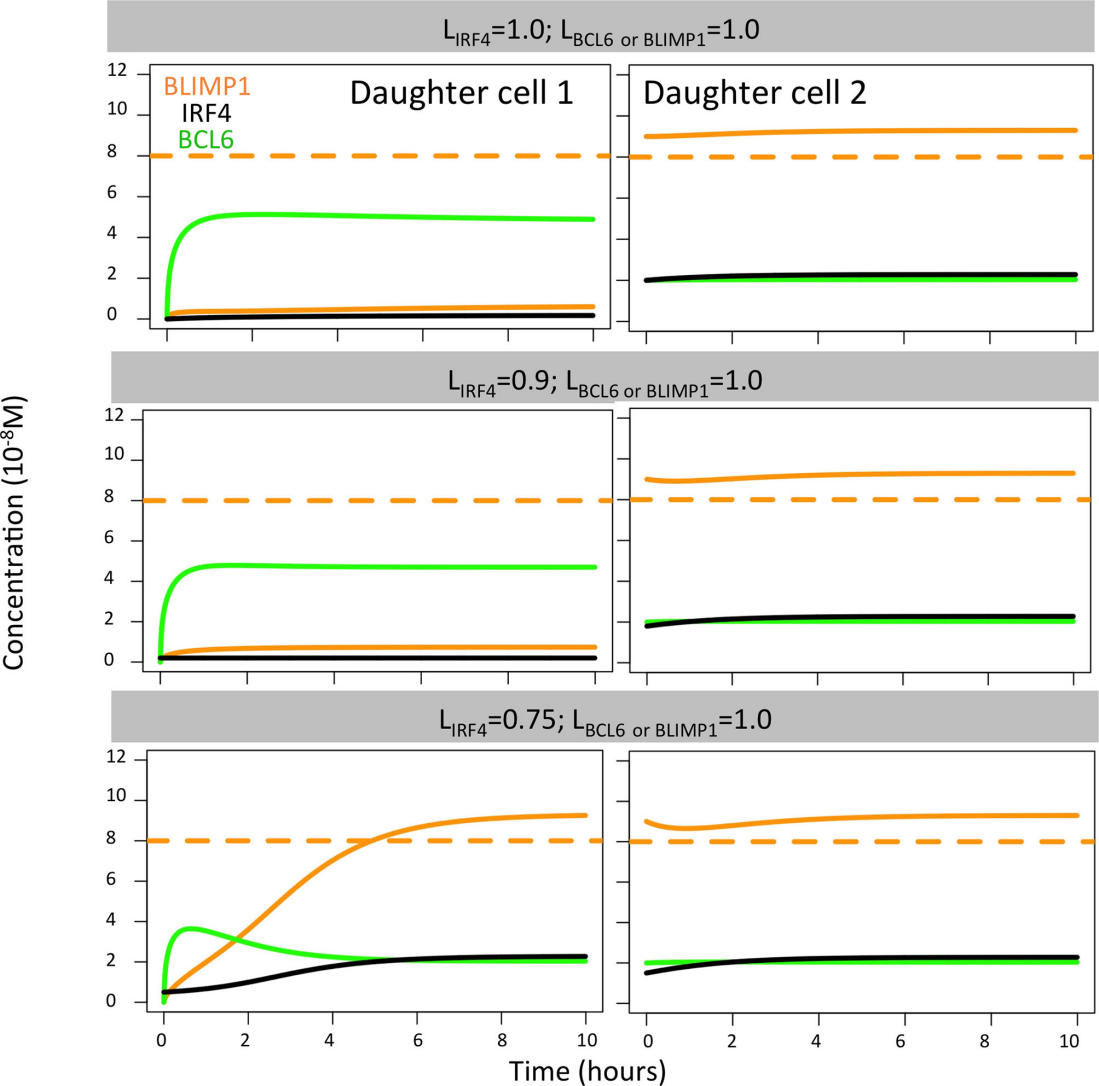
BLIMP1 (orange), BCL6 (green), and IRF4 (black) dynamics in two theoretical daughter B cells. Their initial TF concentrations were set to simulate the asymmetric division of a parent cell (BLIMP1 = 8, BCL6 = 2, IRF4 = 2) with all different combinations of IRF4 levels (L_IRF4_ = 1.0, L_IRF4_ = 0.9, and L_IRF4_ = 0.75, as shown in **Table 2**). Levels of BCL6 and BLIMP1 were fixed (L_BCL6_ = L_BLIMP1_ = 1.0).

When analyzing the TF dynamics in the GRN, we found that extreme IRF4 polarity levels (L_IRF4_ = 1.0, L_IRF4_ = 0.9; L_BLIMP1_ = L_BCL6_ = 1.0) immediately generated a high BLIMP1 state in one of the TF inheriting daughters while leaving the other daughter B cell in a low BLIMP1 state (**Figure 7**). Low IRF4 polarity levels (L_IRF4_ = 0.75; L_BLIMP1_ = L_IRF4_ = 1.0) generated both daughter B cells in the high BLIMP1 steady state. Nevertheless, in this situation, the daughter B cell that inherited 25% (1 − L_IRF4_) of IRF4, along with 0% of BLIMP1 and BCL6 concentration, slowly progressed to the high BLIMP1 state within 20 h until BLIMP1 levels reached the PC differentiation threshold. Considering that after the last division, PBs are defined as PCs and exit the GC, this could explain why no difference in OC dynamics was observed when varying IRF4 polarity levels.

**Figure 8:**
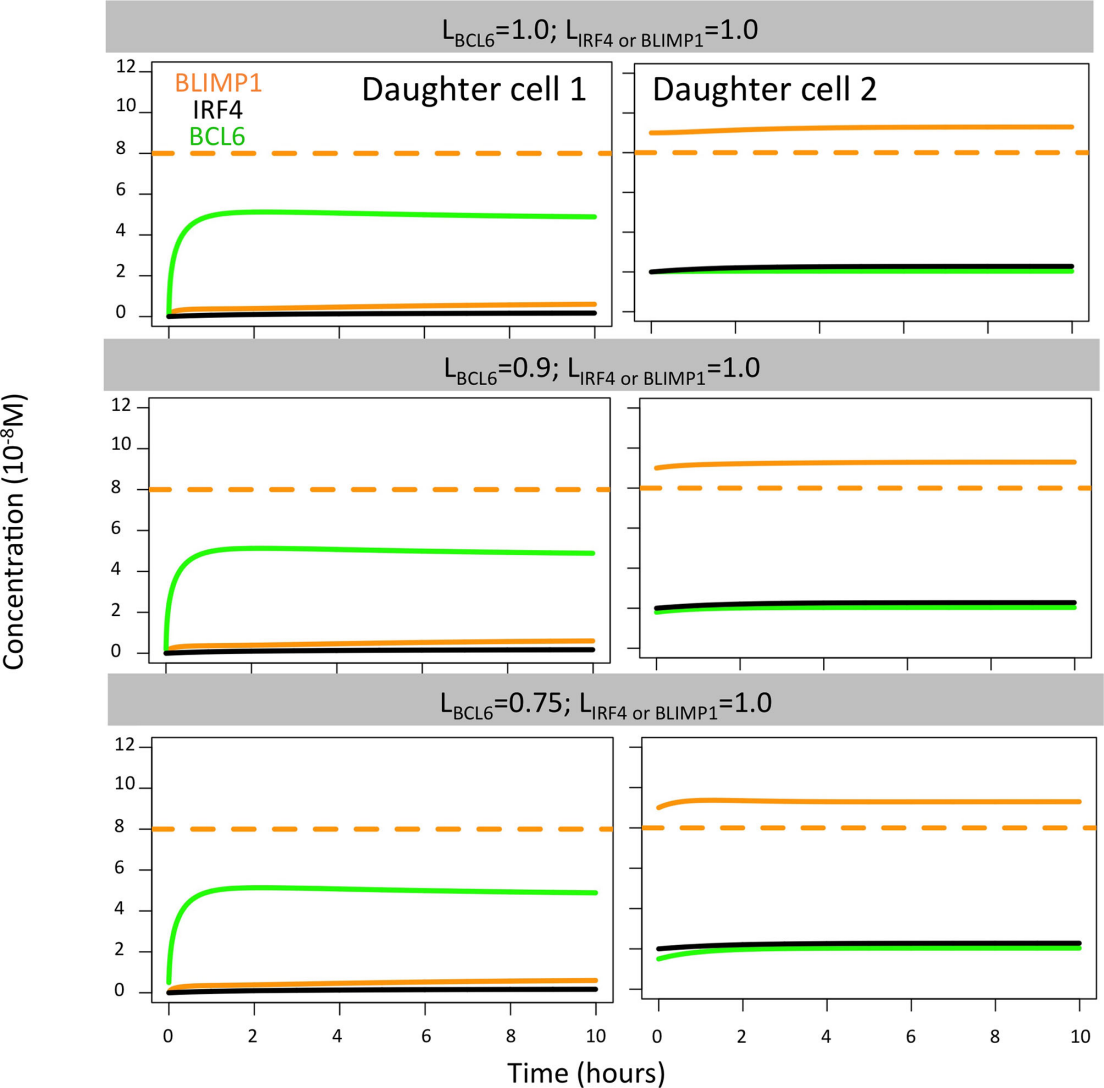
BLIMP1 (orange), BCL6 (green), and IRF4 (black) dynamics in two theoretical daughter B cells. Their initial TF concentrations were set to simulate the asymmetric division of a parent cell (BLIMP1 = 8, BCL6 = 2, IRF4 = 2) with all different combinations of BCL6 levels (L_BCL6_ = 1.0, L_BCL6_ = 0.9, and L_BCL6_ = 0.75, as shown in **Table 2**). Levels of IRF4 and BLIMP1 were fixed (L_IRF4_ = L_BLIMP1_ = 1.0).

In the case of BCL6, we found that all polarity levels (L_BCL6_ = 1.0, L_BCL6_ = 0.9, L_BCL6_ = 0.75; L_BLIMP1_ = L_IRF4_ = 1.0) immediately generated a high BLIMP1 state in one of the TF inheriting daughters, leaving the other daughter B cell in a low BLIMP1 state (**Figure 8**). This is why no difference in OC dynamics was observed when varying BCL6 polarity levels. Such results were not surprising since changes in the BCL6 level as a result of BcR signaling are not sustained in time nor become large enough to switch the BLIMP1 from a high to low level.

**Figure 9:**
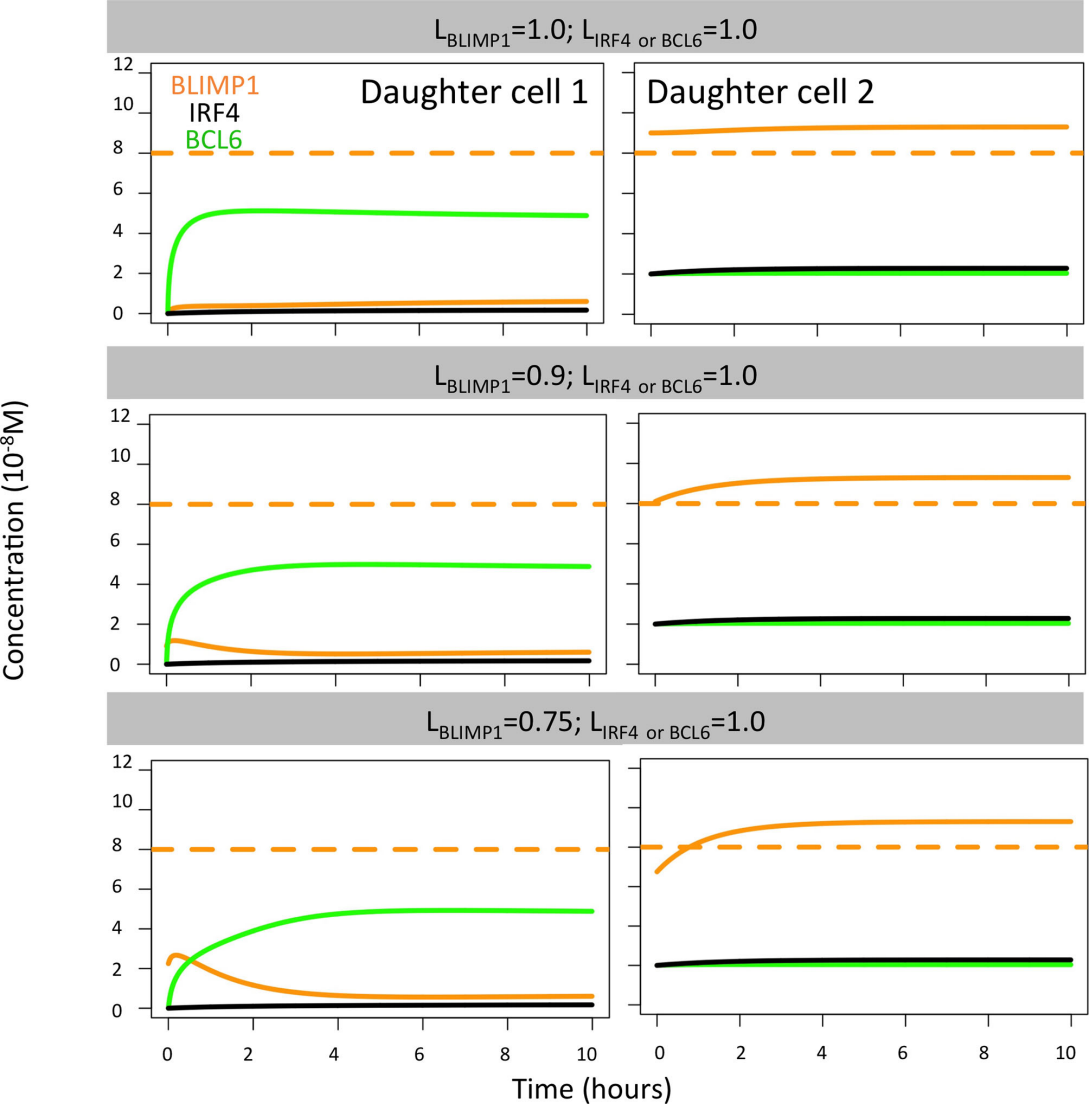
BLIMP1 (orange), BCL6 (green), and IRF4 (black) dynamics in two theoretical daughter B cells. Their initial TF concentrations were set to simulate the asymmetric division of a parent cell (BLIMP1 = 8, BCL6 = 2, IRF4 = 2) with all different combinations of BLIMP1 levels (L_BLIMP1_ = 1.0, L_BLIMP1_ = 0.9, and L_BLIMP1_ = 0.75, as shown in **Table 2**). Levels of IRF4 and BCL6 were fixed (L_IRF4_ = L_BCL6_ = 1.0).

Finally, we found extreme BLIMP1 polarity levels (L_BLIMP1_ = 1, L_BLIMP1_ = 0.9; L_IRF4_ = L_BCL6_ = 1.0) immediately generated a high BLIMP1 steady state in one of the TF inheriting daughters, leaving the other daughter B cell in a low BLIMP1 steady state (**Figure 9**). Low BLIMP1 polarity levels (L_BLIMP1_ = 0.75; L_BCL6_ = L_IRF4_ = 1.0) introduced a delay (4 h) in the progression of the high BLIMP1 inheriting daughter B cell to the high BLIMP1 state. This could explain the differences observed in OC dynamics when varying BLIMP1 polarity levels.

We conclude that the combined results from these 27 simulations and the first set of 9 simulations show that BLIMP1 driven PC differentiation together with coupled asymmetric division of Ag and BLIMP1 with a large segregation between the daughter cells results in a GC DZ-to-LZ ratio and a temporal switch from MBCs to PCs that are both observed in experiments (6, 10) However, future experimental validation of our findings remain necessary.

## Discussion

It has been shown experimentally that Ag and TFs can asymmetrically divide and that this may codetermine GC B-cell fate (3, 5, 7, 8). However, so far, this has not been proven experimentally. Based on a computational model of the GC, Meyer-Hermann and colleagues hypothesized that asymmetric division of Ag might play a role in PC differentiation, as this resulted in a DZ-to-LZ ratio in agreement with experimental data (5). However, using our MSM, we recently showed that asymmetric Ag division alone cannot explain PC differentiation, since it is not fully consistent with experimental observations that B cells with increased BLIMP1 levels differentiate to PCs, but we only considered one specific mode of coupled asymmetric division (i.e., P_Ag_ = P_TF_ = 0.72, L_BLIMP1_ = L_IRF4_ = L_BCL6_ = 1.0) (11). Therefore, in the current work, we investigated the putative effect of asymmetric division of Ag and TFs in more detail and hypothesized that this affects GC dynamics and B-cell dynamics and fate. From our simulations, we conclude that BLIMP1-driven PC differentiation together with coupled asymmetric division of Ag and BLIMP1 with extreme TF polarity levels for BLIMP1 segregation results in GC DZ-to-LZ ratio and a temporal switch from MBCs to PCs that are also observed in experiments (6, 10). This confirmed our previous finding that asymmetric Ag division alone is not sufficient to drive PC differentiation, but also asymmetric division of at least BLIMP1 is required.

An important insight from our model is the observation that outcomes of simulations with (uncoupled) symmetric division of Ag and/or TF do not agree with experimental observations (migrations rates, temporal switch). It is, however, important to emphasize that this result does not definitely exclude this scenario to be true. Although our GC model is the most sophisticated model currently available and based on a large range of experimental observations, we cannot exclude the possibility that other choices, assumptions, or parameter settings would change our conclusion. Nevertheless, we think that our simulations provide at least some evidence that asymmetric division is involved in PC differentiation. Furthermore, prior studies have shown that unequal stimulation of signaling pathways, e.g., CD40 and PI3K, induced when B cells present Ag to and receive help from T_FH_ cells during the selection process in the GC reaction, can provide polarity cues that drive asymmetry division (7, 8). It was proposed that unequal inheritance of Ag transmembrane receptor, costimulation, and/or cytokine signaling could result in unequal activation of signaling pathways. Although this hypothesis was not experimentally tested, it is in line with our finding.

The observation that IRF4 asymmetric division had no effect of PC production was both interesting and surprising. On the one hand, *in vitro* data suggest that IRF4, and/or different levels of T help through Cd40/Nf-kB induction of IFR4, regulates MBC and PC differentiation in a concentration-dependent manner (13, 14). Furthermore, quantitative modeling of the terminal B-cell differentiation showed through parameter sensitivity analysis for bistability that kinetic parameters associated to IRF4 dynamics and CD40 induction of IRF4 were critical in promoting B-cell transition towards PC differentiation (12). Nevertheless, the same study showed that above a critical IRF4 concentration threshold (>1.10^−8^M), CCs irreversibly differentiated to PCs. In our model, asymmetric division takes place at a late stage of B-cell development (PB) in which IRF4 concentration is close to the high IRF4 steady state (2.10^−8^M). Thus, we found that even with low IRF4 polarity levels, when daughter B cells inherited 75% of IRF4 (L_IRF4_ = 0.75), this did not decrease IRF4 concentration below the above-mentioned critical IRF4 threshold. This explained why we found no effect of IRF4 asymmetric division on PC differentiation. In addition, *in vitro* studies in conjoined sibling B cells showed that unequal IRF4 expression could drive branching of B-cell state prior to the loss of PAX5, a MBC promoter, hence at early stages of B-cell transition to PC. Furthermore, the levels of BLIMP1 in sibling B cells were not measured, leaving the open question of whether asymmetric BLIMP1 division could be the driver of PC differentiation and supporting the need to further investigate BLIMP1 asymmetric division at later stages of PC differentiation in the GCs.

Apart from model assumptions, our study has several limitations. First, our findings and conclusions remain to be validated or falsified in future experiments. We propose experiments to generate data about the (1) BLIMP1 probability of asymmetric division and polarity level in single PBs; (2) extent and/or role of the cosegregation of BLIMP1, BCL6 and IRF4; and (3) extent and/or role of (a)symmetric division of CD40 signaling in relation to B-cell fate. Second, the probability (P_Ag_ = 0.72) for asymmetric Ag division was based on experimental data (7). For asymmetric TF division, we used this same value in several simulations. However, probabilities of P_BCL6_ = 0.44 and P_IRF4_ = 0.11 have been reported (7), while for BLIMP1, such probability is unknown. Nevertheless, we here show that asymmetric division of IRF4 and BCL6 did not have an effect on the fate of the B cell, and thus, we believe that this would not change our main conclusion. Third, no data are available about the number of MBCs and PCs produced during a single GC reaction. Thus, we cannot substantiate which simulations are more realistic in terms of OC production. Fourth, as we have discussed previously (11), the current definition of MBCs as Ag+BLIMP1− cells should be improved, since it definition merely classifies OCs, which are not PCs to be MBCs. Nevertheless, we here showed that symmetric TF division did not agree with the observation of a temporal switch in the GC reaction. This could indicate that asymmetric TF division plays a role in MBC differentiation. Interestingly, PAX5 has been shown to asymmetrically segregate and always oppose asymmetric IRF4 distribution (8). Further experiments need to be carried out to validate this hypothesis since the effect of asymmetric PAX5 division on MBC formation was not addressed.

## Data Availability Statement

Publicly available datasets were analyzed in this study. This data can be found here: https://github.com/EDS-Bioinformatics-Laboratory/AsymmetricDivision.

## Author Contributions

EM, HH, and AK designed the study. EM, DL, MM-H, and PR implemented the software. JH, EM, DL, and AK carried out the simulations and analyses. All authors were involved in the interpretation of results. JG, HH, and AK supervised the study. All authors contributed to the article and approved the submitted version.

## Funding

This work is supported by a CASyM Exchange Research Grant, COSMIC (www.cosmic-h2020.eu), which has received funding from the European Union’s Horizon 2020 research and innovation programme under the Marie Skłodowska-Curie grant agreement No 765158, and by the Human Frontier Science Program 570 (RGP0033/2015).

## Conflict of Interest

The authors declare that the research was conducted in the absence of any commercial or financial relationships that could be construed as a potential conflict of interest.

## Publisher’s Note

All claims expressed in this article are solely those of the authors and do not necessarily represent those of their affiliated organizations, or those of the publisher, the editors and the reviewers. Any product that may be evaluated in this article, or claim that may be made by its manufacturer, is not guaranteed or endorsed by the publisher.
