# Supplementary material for: Coupled Antigen and BLIMP1 Asymmetric Division With a Large Segregation Between Daughter Cells Recapitulates the Temporal Transition From Memory B Cells to Plasma Cells and a DZ-to-LZ Ratio in the Germinal Center
Source: Front Immunol. 2021 Aug 17;12:716240. doi: 10.3389/fimmu.2021.716240 (PMC8416073; doi:10.3389/fimmu.2021.716240)
Supplement: Supplementary file 1 [file DataSheet_1.docx]

**Supplementary Information**


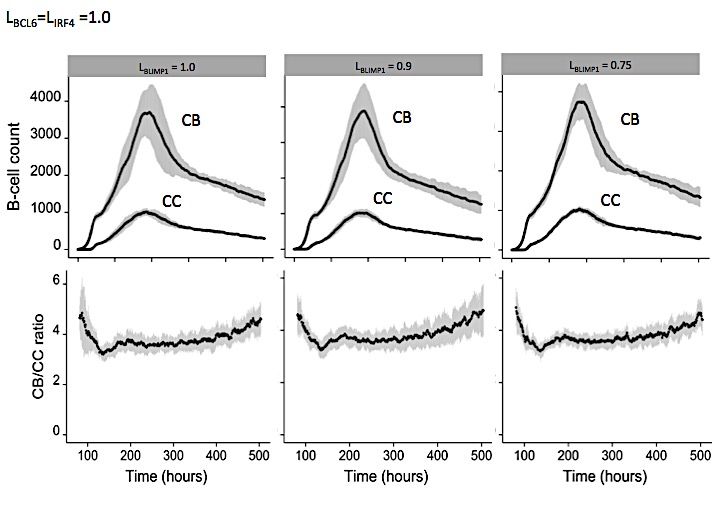


**Supplementary Figure 1:** Overall GC dynamics for three BLIMP1 polarity levels for Simulations 1-3 (Table 2). (Top) CB and CC counts. (Bottom) DZ-to-LZ ratio. Mean and standard deviation from of 15 different repetitions (random seeds) are shown.


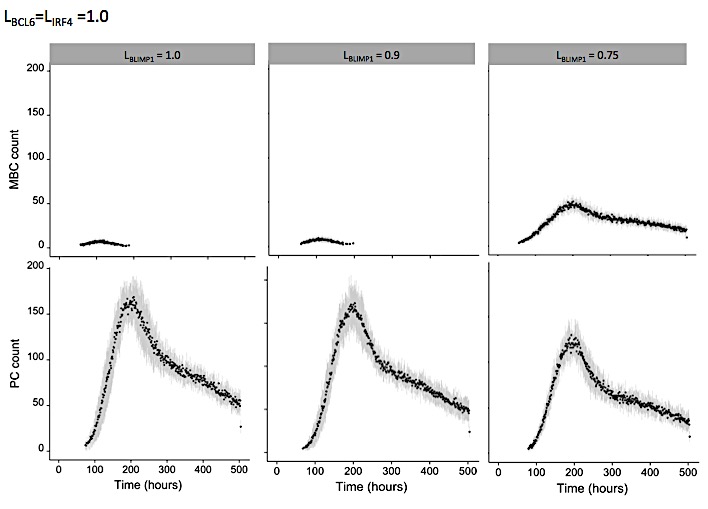


**Supplementary Figure 2:** Overall MBC and PC dynamics for three BLIMP1 polarity levels for Simulations 1-3 (Table 2). (Top) MBC counts. (Bottom) PC counts. Mean and standard deviation of 15 different repetitions (random seeds) are shown.


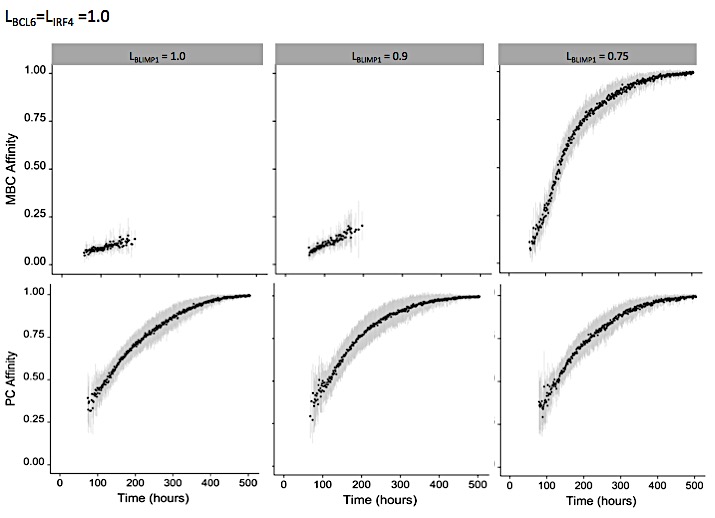


**Supplementary Figure 3:** Overall MBC and PC affinity dynamics for three BLIMP1 polarity levels for Simulations 1-3 (Table 2). (Top) MBC mean affinity. (Bottom) PC mean affinity. Mean and standard deviation of 15 different repetitions (random seeds) are shown.


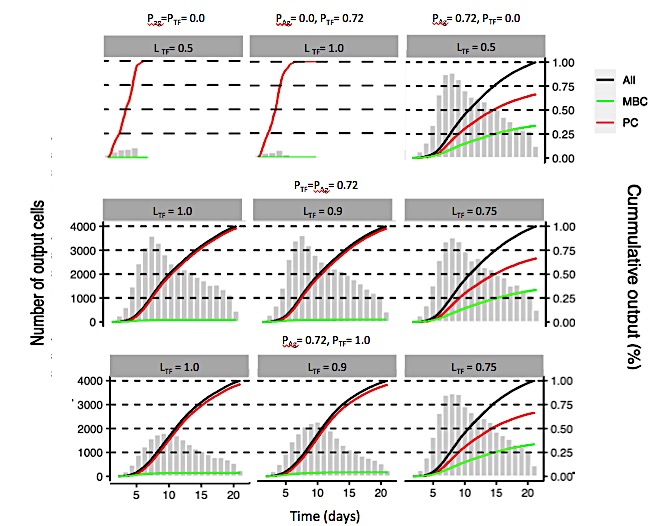


**Supplementary Figure 4:** (Cumulative) number of OCs. Histogram represents the number of OCs per day for Simulations 1-9 (Table 2). Black, red and green lines represent the cumulative percentage of output, PCs and MBCs respectively.

**Supplementary Figure 5:** (Cumulative) number of OCs. Histogram represents the number of OCs per day for Simulations 1-9 (Table 3). Black, red and green lines represent the cumulative percentage of output, PCs and MBCs respectively.

**Supplementary Figure 6:** (Cumulative) number of OCs. Histogram represents the number of OCs per day for Simulations 10-18 (Table 3). Black, red and green lines represent the cumulative percentage of output, PCs and MBCs respectively.

**Supplementary Figure 7:** (Cumulative) number of OCs. Histogram represents the number of OCs per day for Simulations 19-27 (Table 3). Black, red and green lines represent the cumulative percentage of output, PCs and MBCs respectively.
